# Neoantigens Generated by Individual Mutations and Their Role in Cancer Immunity and Immunotherapy

**DOI:** 10.3389/fimmu.2017.01679

**Published:** 2017-11-28

**Authors:** Mirjana Efremova, Francesca Finotello, Dietmar Rieder, Zlatko Trajanoski

**Affiliations:** ^1^Biocenter, Division of Bioinformatics, Medical University of Innsbruck, Innsbruck, Austria

**Keywords:** next-generation sequencing, immunoediting, tumor heterogeneity, somatic mutations, cancer vaccines

## Abstract

Recent preclinical and clinical studies have proved the long-standing hypothesis that tumors elicit adaptive immune responses and that the antigens driving effective T-cell response are neoantigens, i.e., peptides that are generated from somatically mutated genes. Hence, the characterization of neoantigens and the identification of the immunogenic ones are of utmost importance for improving cancer immunotherapy and broadening its efficacy to a larger fraction of patients. In this review, we first introduce the methods used for the quantification of neoantigens using next-generation sequencing data and then summarize results obtained using these tools to characterize the neoantigen landscape in solid cancers. We then discuss the importance of neoantigens for cancer immunotherapy using checkpoint blockers, vaccination, and adoptive T-cell transfer. Finally, we give an overview over emerging aspects in cancer immunity, including tumor heterogeneity and immunoediting, and give an outlook on future prospects.

## Introduction

In the past decade, driven by technological advances major progress in cancer research and cancer therapy was made. First, the development of next-generation sequencing (NGS) technologies and large-scale projects such as The Cancer Genome Atlas (TCGA) resulted in a comprehensive characterization of the human cancer genomes. And second, novel drugs targeting immune checkpoint molecules have been approved in several malignancies and are showing remarkable clinical effects. These drugs augment T-cell activity by blocking cytotoxic T lymphocyte antigen-4 (CTLA-4), programmed cell death protein 1 (PD-1), or PD-1 ligand. Long-term data of patients who received anti-CTLA-4 antibodies in unresectable or metastatic melanoma indicate curative potential in a fraction of patients (1). Moreover, efficacy of anti-PD-1 antibodies has been shown not only in melanoma, but in an increasing number of other cancers (2). Not surprisingly, there are now enormous efforts for the development of novel immunotherapeutic strategies with over 1,000 clinical trials with monotherapies or combination therapies (3).

One specific advantage of cancer immunotherapy is the potential to adapt to the evolution of the tumor since specific T cells can develop which are targeting newly developed tumor clones. T cells recognize tumor-specific antigens bound to the major histocompatibility complex (MHC) molecules of tumor cells. Antigens with high tumoral specificity have the potential to elicit tumor-specific immune responses and are, therefore, of great interest for cancer immunotherapeutic strategies, including therapeutic vaccines and engineered T cells. There are three classes of antigens with high tumoral specificity: (1) viral antigens that are derived from genes expressed in virus-infected tumor cells; (2) cancer-germline antigens, also known as cancer-testis antigens. These are proteins that are expressed only by germline cells and have aberrant expression in tumor cells; and (3) neoantigens, i.e., are peptides that are generated from somatic mutations. During tumor progression, mutations accumulating in the tumor genome can affect protein-coding genes and result in altered protein sequences. Mutated proteins are proteolytically cleaved into short peptides and presented on the tumor cell surface by the MHC—called human leukocyte antigen (HLA) in humans. These mutated neoantigens, which are present in the malignant cells but not in the normal cells can be recognized as foreign by tumor-infiltrating lymphocytes (TILs) and elicit potent tumor-specific immune responses. Neoantigens released after tumor cell death initiate a number of processes that ultimately lead to T cells that recognize cancer cells through the interaction of distinct T-cell receptors (TCR) with specific neoantigen–MHC complexes.

The tumor–immune cell interaction can be conceptualized as a number of processes conceptualized as the cancer-immunity cycle (4). The first step in this cycle is the generation of neoantigens (neoepitopes) and, therefore, the identification and characterization of neoantigens is of utmost importance for deriving novel mechanistic insights on cancer immunity and developing efficient cancer immunotherapies. In this review, we give an overview of the current advances in the computational prediction of neoantigens and discuss the development of cancer immunotherapies targeting neoantigens, including vaccination, checkpoint therapy, and adoptive cell transfer.

## Quantifying Neoantigens Using NGS Data

Neoantigens can be experimentally determined using proteomic analysis of MHC ligands by liquid chromatography and tandem mass spectrometry (5–7). However, this approach is labor intensive and requires large amount of material for the analysis, which is seldom available from human biopsies. Alternatively, when NGS data are available from matched tumor and normal samples, neoepitopes can be predicted by integrating four computational tasks: (i) prediction of somatic DNA mutations; (ii) identification of mutated proteins; (iii) *in silico* HLA typing; and (iv) selection of the mutated peptides with high binding affinity to the predicted MHC/HLA molecules and high expression of the mutation-encoding gene [see recent comprehensive review (8)]. Somatic DNA mutations are usually computed from whole-exome (WES) or whole-genome sequencing (WGS) data from matched tumor-normal samples using computational tools for variant detection, and can be further processed with software for variant annotation to predict the affected proteins (9). Patient-specific NGS data from WES, WGS, or RNA sequencing (RNA-seq) can be also used to predict HLA types with computational tools like Polysolver (10) and Optiptype (11), which are able to extract the reads covering the HLA locus and predict the major alleles at 4-digit resolution or more. Finally, machine learning algorithms such as NetMHCpan (12) trained on experimental data can be used to predict which short peptides spanning protein regions affected by mutations bind with high affinity to the predicted HLA types.

The single tools performing the three computational tasks described above require a number of intermediate steps for data preprocessing and formatting which is usually carried out in specialized bioinformatics labs. In order to broaden the utility of the computational genomics tools, a number of computational pipelines that integrate the individual steps were recently developed. Such pipelines for *in silico* prediction of personalized neoantigens from NGS data with different degrees of functionality include pVAC-seq (13), FRED 2 (14), INTEGRATE-neo (15), and MuPEXI (16). However, although an improvement to the use of individual steps, assembling analytical pipelines and executing workflows with a number of consecutive steps is laborious and depends on many parameter settings. The recently developed pipeline TIminer (17) integrates cutting-edge bioinformatics tools for the analysis of both, RNA-seq data and somatic DNA mutations in order to characterize the tumor–immune interface. This pipeline enables: (1) genotyping of HLAs using exome-sequencing or RNA-seq data, (2) prediction of tumor neoepitopes using specific HLA types and mutations, and (3) characterization of TILs from bulk RNA-seq data.

The available computational pipelines predict neoepitopes that bind to class-I MHC molecules. Peptides binding to class-I MHC molecules, which exist on almost all nucleated cells, are presented for recognition by cytotoxic CD8^+^ T cells. Class-II MHC molecules are present only on professional antigen-presenting cells, such as dendritic cells, macrophages, and B lymphocytes, and display antigens to CD4^+^ helper T cells. Although coordinated CD4^+^ and CD8^+^ responses are required for tumor control and rejection, the suboptimal performance of the current algorithms for prediction of class-II neoantigens limits their translational potential for personalized cancer medicine. The need for better methods for prediction of class-II neoantigens has increased ever since studies showed that CD4^+^ T cells recognize a higher number of neoantigens than was previously known and can generate potent antitumor response (17). More recently, a proof-of-concept by Sahin et al. and Ott et al. using a combined strategy for class-I and class-II neoantigen prediction was presented (18, 19).

There are several challenges with MHC–peptide-binding prediction algorithms. First, experimental data from measurements of the biochemical affinity of synthetic peptides, needed for the training of these algorithms, are limited for MHC class-II alleles. Therefore, while effective in predicting many epitopes, these approaches may nevertheless be limited in their accuracy due to the sparsity of both positive and negative training data sets and result in high false-positive rate. For example, in Robbins et al., 229 tumor-specific neoepitopes were predicted across three melanoma patients, but only 11 of these neoepitopes elicited a T-cell response (20). In addition, these methods do not necessarily consider the endogenous processing and transport of peptides prior to HLA binding. In order to improve neoantigen predictions, Abelin et al. developed a new biochemical and computational pipeline for LC–MS/MS analysis of endogenously processed HLA-associated peptides along with a predictor that outperformed current algorithms that are trained on peptide affinity data (21).

## Neoantigen Landscape in Solid Cancers

Given the availability of NGS data from cancer samples from large-scale projects such as the TCGA, as well as the improved performance of the computational tools, a number of studies analyzed neoantigens and association with clinical parameters and molecular entities. A seminal work by Holt and colleagues showed an association between neoantigen load and survival (22). We recently generated high-resolution maps on neoantigens and the immunophenotypes in colorectal cancer (CRC) (23) using genomic data sets from the TCGA cohort (*n* = 598) (24). The neoepitopes were barely shared between patients: only 4% of the predicted neoepitopes were shared between 2 and more patients. The shared neoepitopes are identical peptides that originate from one or more genes. Importantly, we observed that the genetic basis of the tumors determines the tumor escape mechanisms. For example, hypermutated tumors had higher intratumor heterogeneity, indicating that the larger mutational load results not only in a larger neoantigen load but also in a more diverse neoepitope landscape, and therefore likely promotes T-cell activation and infiltration.

**Figure 1:**
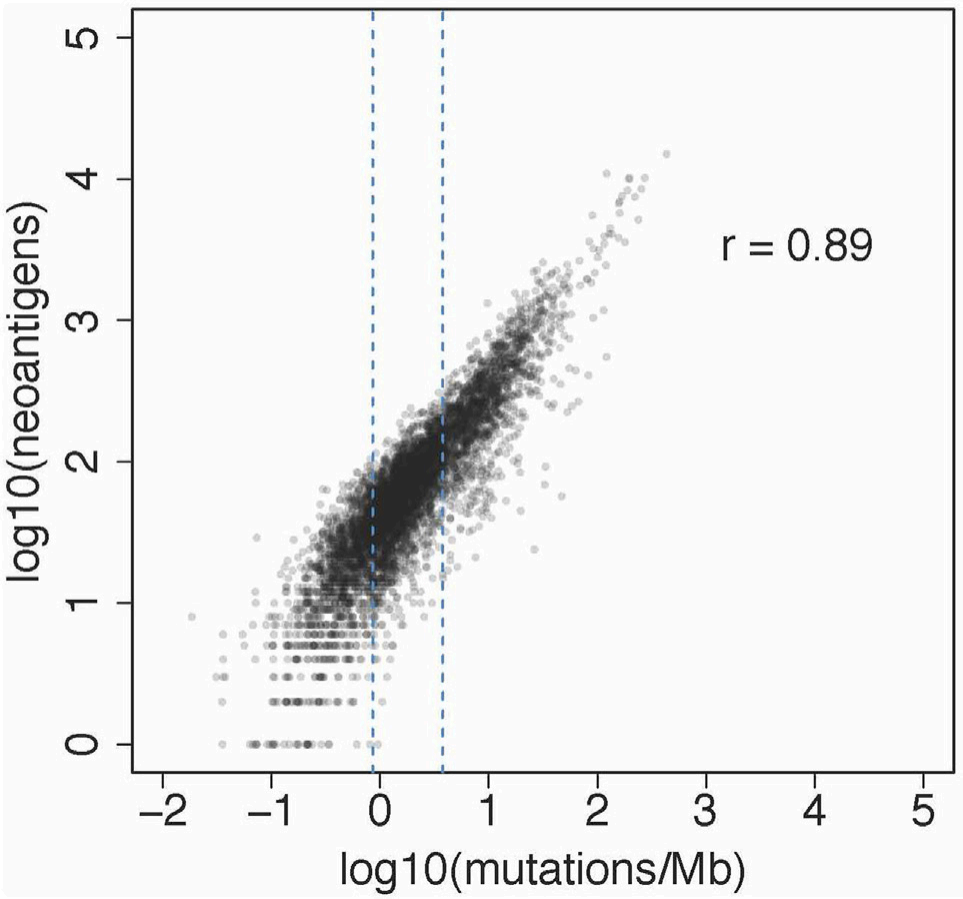
Association between neoantigens and mutations from a pan-cancer analysis reported recently (25). The plot shows the results of the analysis of 6,726 patients from 19 solid cancers. The number of neoantigens per subject ranged from 1 to 15,035 and the number of mutations per MB per patients from 0.019 to 933.085.

We then extended the analysis and characterized more than 8,000 patients from the TCGA comprising 19 solid cancers (25) (results available at https://tcia.at). As expected, our pan-cancer analysis showed that the number of neoepitopes correlated with the mutational load. The results of this analysis are shown in Figure 1. Moreover, the number of neoepitopes correlated also with the infiltration of TILs. The fraction of neoepitopes generated from driver genes was 7.6%. Thus, the majority of neoepitopes had its origin in passenger genes. Similar to the CRC study, the results showed that the neoepitopes were seldom shared. From the total of 911,548 unique predicted neoepitopes, only 24 were common in more than 5% of patients. As expected, the most frequent predicted neoepitopes were induced by mutations in driver genes, such as BRAF, RAS, and PIK3CA. Thus, the results show that the neoantigen landscape in solid cancers is not only highly diverse both, between and within cancers, but also extremely sparse. The sparsity of the neoantigen space clearly argues against vaccination strategy based on off-the-shelf vaccines. Rather, cancer vaccination strategies based on neoepitopes has to be personalized. This can be achieved by using whole-exome NGS for the identification of somatic mutations and bioinformatic prediction of neoepitopes, followed by synthesis of peptide- or DNA/RNA-based vaccines. Proof of concept for this type of individualized cancer vaccination was recently shown in several clinical studies (18, 19, 26).

## Neoantigens and Cancer Immunotherapy

Self-antigens that are aberrantly expressed in cancerous tissues can provoke an immune response and have been used in the past in clinical studies. However, expression of these antigens in normal tissues can initiate central and peripheral tolerance mechanisms. Lately, more efforts have been focused on antigens derived from mutated proteins. Since T cells recognizing neoantigens are not influenced by central immune tolerance because of the lack of expression in healthy tissues, targeting of tumor neoantigens may be more specific and less toxic than other approaches, making neoantigens attractive targets for immunotherapy, including therapy with antibodies directed against immune checkpoint blockers, therapeutic vaccination, or adoptive T-cell transfer with TCR-engineered neoantigen-specific T cells.

### Neoantigens and Response to Therapy with Checkpoint Blockers

An increasing number of studies have shown a strong association of the mutation/neoantigen burden with TIL infiltration and activity, as well as better response to therapy and overall survival in non-small cell lung cancer (NSCLC) and melanoma patients (27–29). Both types of cancers accumulate high number of mutations as a result of exposure to mutagens, such as tobacco smoke and ultraviolet light. Tumors with microsatellite instability due to deficiency in the mismatch repair system show high mutational burden, T-cell infiltration, improved survival, and durable clinical benefit when treated with checkpoint blockers. Similarly, tumors with mutations in other DNA repair pathways (30–32) showed an enhanced T-cell response and better response to checkpoint blockers. A recent study by Le and colleagues provided further evidence of the sensitivity of mismatch-repair-deficient cancers to checkpoint blockade, irrespectively of the tissue of origin (33). The authors evaluated the efficacy of anti-PD-1 treatment in patients with mismatch repair deficiency from 12 different cancer entities and reported objective responses in 53% and complete responses in 21% of these patients. Moreover, they demonstrated through functional analysis in a responding patient that tumor-reactive lymphocytes were directed against mutated neoantigens. As a comparison, MSS CRC and cancers with low mutational load such as prostate cancer have shown little or no benefit from immunotherapies, providing additional evidence for the importance of neoantigens in the antitumor immune response. However, there are also cases of cancers with high neoantigen burden showing no response to immune checkpoint therapies [e.g., 50% of the microsatellite instable (MSI) cancers], as well as cancers characterized by low neoantigen load that are susceptible to immunotherapy (34). Thus, it can be argued that the high neoantigen load represents merely a higher likelihood of the presence of immunogenic neoantigen suggesting that neoantigen landscape alone is not sufficient in predicting immunotherapy responses.

### Cancer Vaccination

Individualized vaccines, designed to present neoantigens to prime and activate dendritic cells have also been used to selectively target neoantigens. Vaccines have been shown to both expand pre-existing neoantigen-specific T-cell populations as well as broaden the TCR repertoire. In addition to enhancing the strength and persistence of T cells, vaccines can elicit an immune response for neoantigens that were undetectable prior to vaccination (26). Therefore, even if the neoantigens that spontaneously induce T-cell responses are lost due to immunoediting, the neoantigens that do not naturally elicit a response can serve as targets for vaccines (35). Two recent studies (18, 19), used vaccines based on neoantigens recognized by CD4^+^ and/or CD8^+^ T cells to demonstrate that personal neoantigen vaccines, alone or in combination with checkpoint blockade, can induce both effective and safe immune response. The authors reported T-cell infiltration induced by vaccination and specific killing of tumor cells expressing neoantigens. Ott et al. used synthetic long 15–30-mers peptides and immunized six melanoma patients, two of which had lung metastases (35). Four out of six patients had no disease recurrence, whereas metastatic patients were further treated with anti-PD-1 therapy and showed complete tumor regression. Sahin et al. used an RNA-based approach using predicted neoantigens recognized by CD4^+^ and CD8^+^ T cells in 13 melanoma patients (18). Neoantigen-based vaccination reduced metastatic events and caused objective response in two over five metastatic patients and, even more strikingly, a complete response in a third patient treated with the vaccine in combination with anti-PD-1 immunotherapy. Both reports further indicate that neoantigens are important targets for mediating response to checkpoint therapies and, additionally, that the tumor-reactive T cells target diverse tumor clones, thereby dealing with extensive tumor heterogeneity.

### Adoptive T Cell Transfer

Neoantigens can be used to expand neoantigen-specific T cells *in vitro* for use in adoptive T-cell transfer. Several studies reported tumor regression achieved by transfer of autologous TILs resected from patients with metastatic melanoma (20, 36). The Rosenberg group demonstrated dramatic tumor regression in a metastatic cholangiocarcinoma patient treated with a personalized adoptive cell transfer, where over 95% of autologous T cells consisted of CD4^+^ T cells that recognized a single HLA class-II-restricted neoantigen (37). They also demonstrated the therapeutic efficacy of neoantigen-specific CD4^+^ T cells. In a study by van Rooij et al., in which exome sequencing and MHC tetramer screening was used, compelling evidence was provided that immunotherapy with checkpoint blockers in a melanoma patient induces expansion of already existing T cells which are targeting neoantigens (38).

Despite the success of immunotherapies targeting neoantigens, many questions remain unanswered. To begin with, only few of the predicted neoantigens elicit potent immune responses. Even though only a small percentage of the *in silico* predicted neoantigens are actually immunologically recognized, Strønen et al. provided evidence of the existence of a neglected neoantigen repertoire that induces T-cell reactivity and may broaden neoantigen-specific T-cell reactivity and enable the targeting of neoantigens that have not been recognized by the patient’s own immune system (39). This is further supported by two other studies (18, 35) that showed that responses against neoantigens can be induced *de novo*. One possible explanation for the hidden neoantigen repertoire is the immunodominance of tumor antigens: the immune system targets particular tumor antigens but ignores others (40), a phenomenon that often occurs with viral antigens. In addition, lack of T-cell priming against tumor-associated antigens can result in the exclusion of T cells from the tumor microenvironment. For example, Spranger et al. (39) reported that oncogenic WNT/β-catenin signaling pathway prevents T-cell and CD103^+^ DC infiltration in melanoma and generates resistance to checkpoint blockade therapy.

In conclusion, genomic approaches can facilitate the development of personalized immunotherapies directed at neoantigens. Therapeutic stimulation of broad neoantigen-specific T-cell responses through vaccines targeting multiple antigens could help overcome the effects of tumor heterogeneity as well as avoid resistance, by providing broader coverage of the whole tumor cell population. As highly homogeneous tumors have been shown to be more immunogenic and since clonal neoantigens seem to drive antitumor responses following therapy with antibodies against immune checkpoints, a potential approach is to target clonal neoantigens, i.e., those that are expressed in all tumor cells within a patient, in order to overcome the significant challenge posed by intratumor heterogeneity. However, different cancers undergo different evolutionary trajectories: some are dominated by Darwinian selection pressures that shape their clonal composition, whereas others follow neutral evolution. In order to successfully target the whole tumor population and prevent escape of resistant clones, comprehensive genomic and immunogenomic analyses of pre- and post-treatment samples are needed for longitudinally evaluating changes in the tumor. Ultimately, a deeper understanding of the evolutionary and immune-related forces that shape the tumor progression will be fundamental to improve the efficacy of immunotherapy and minimize resistance.

## Tumor Heterogeneity, Immunoediting, and Acquired Resistance to Cancer Immunotherapy

### Tumor Heterogeneity

Mutational processes and genomic instability can result in extensive tumor heterogeneity, which has important clinical and immunological implications. While it is well established that intratumoral heterogeneity has an impact on the response of cancer patients to treatments with targeted therapies (41–43), the role of the immune surveillance and sensitivity of the tumors to therapy with checkpoint blockers are only beginning to emerge. Recent studies provided insights into the effect on intratumoral heterogeneity on the immune response and showed that the mutational load in combination with the intratumoral heterogeneity is a better predictor of response to checkpoint blockers than the neoantigen burden alone. More homogeneous tumors and tumors with high clonal neoantigen burden have been associated with higher T-cell infiltration, better prognosis, and better sensitivity to immunotherapeutic approaches (44–46). McGranahan et al. demonstrated that tumors from melanoma and NSCLC patients enriched with clonal neoantigens displayed an inflamed phenotype and were more sensitive to checkpoint blockade therapy (44). These findings raise the question whether immunotherapy will be also effective in heterogeneous tumors as the targeting of subclonal neoantigens by cytotoxic T cells is not sufficient to eradicate the whole tumor.

Although intratumoral heterogeneity presents a challenge for conventional and targeted therapies, increased mutational diversity may provide a beneficial opportunity for immunotherapies by generating potential neoantigens that can be recognized by T cells (47). Very high intratumoral heterogeneity has also been correlated with better prognosis, implying a possible trade-off between acquiring an immunogenic mutation that can elicit an immune response or a driver mutation that can confer a fitness advantage to the tumor cells (45). Highly heterogeneous tumors are possibly driven by neutral evolution, resulting into many subclonal mutations with little or no impact on cancer progression, but potentially generate neoantigens able to elicit an immune response (48).

### Cancer Immunoediting and Acquired Resistance to Immunotherapy

The cancer immunoediting hypothesis postulates a dual role of immunity in the complex interaction between the tumor and the host: the immune system, by recognizing tumor-specific antigens, not only can protect the host by eliminating tumor cells but can also sculpt the developing tumor by editing the cancer genome, and thereby modifying the heterogeneity of the tumor. Strong immunoediting would render tumors more homogeneous by eradicating immunogenic clones (49). Elimination of neoantigens by a T cell-dependent selection process has been suggested as a mechanism of cancer immunoediting in mouse models and human studies (50). Experimental evidence from mouse models and human studies now provides strong support for the existence of cancer immunoediting in many cancers.

The definitive work supporting the existence of cancer immunoediting was published in 2001 by the Schreiber lab and showed that immunodeficient Rag2^−/−^ mice develop spontaneous and carcinogen-induced tumors more rapidly and more frequently than genetically matched wild-type controls (51). Moreover, the tumors arising in immunodeficient animals were frequently rejected following transplantation into immunocompetent recipients, however, when implanted into secondary immunodeficient hosts the effects were not observable. Hence, it seems that tumors from those mice were more immunogenic compared to the tumors from wild-type mice. In a more recent study, cancer immunoediting was investigated in the same mouse model of sarcoma using NGS of the tumor exome and algorithms for predicting neoantigens (52). Their results demonstrated that MCA tumors with a mutant form of spectrin β2 (also known as SPTBN1) were rejected, whereas other tumors developed because of a T-cell-dependent selection of tumor cells that lacked expression of the spectrin β2 antigen. Similar observations were obtained using an oncogene-driven model of cancer in genetically engineered, immunodeficient mice (53) in which primary sarcomas were edited through selection of cells that either did not express antigens or were unable to present antigens to T cells. These studies demonstrated that dynamic interactions between tumors cells and T cells lead to immunoediting. In contrast to carcinogen-induced highly mutated tumors, non-immunogenic tumors with low neoantigen burden do not undergo spontaneous immunoediting (54). In addition, longitudinal samples of pre- and post-treatment samples have shown that different therapies also impose strong selective pressure that can affect the tumor clonal architecture and change the evolutionary path of tumor progression. For instance, patients with a high number of subclonal mutations due to treatment with an alkylating agent were reported to have a poor response to anti-CTLA-4 therapy (44).

Even though immunoediting is more difficult to study in humans, there have been several studies exploring the neoantigen dynamics over time and before and after therapy in patients. A pan-cancer study of TCGA patients in which observed and expected numbers of neoepitopes were analyzed provided the first evidence of immunoediting in human cancers (28). The authors showed that neoantigens are depleted in some cancer types relative to their expected numbers, indicating immune-mediated elimination of tumor subclones that contain neoantigens. Using a similar approach, we recently provided additional data that support the existence of immunoediting in MSI CRC (55).

More recent studies explored the evolution of the neoantigen landscape over time and in response to therapy-induced immune editing. Verdegaal et al., using longitudinal samples from two melanoma patients treated by adoptive T-cell transfer, observed loss of the mutant allele in two cases and reduced expression of T-cell-recognized neoantigens in another one, suggesting potential T-cell dependent selection of antigen-negative variants (56) However, they additionally reported an increased expression of one mutated gene over time. Anagnostou et al. analyzed matched pretreatment and resistant tumors in patients with NSCLC that acquired resistance following a response to therapy targeting PD-1 and/or CTLA-4 (57). The authors identified immunogenic neoepitopes that were not detectable in the resistant tumors due to an elimination of tumor subclones or chromosomal deletions, and proposed therapy-induced immunoediting of neoantigens as a mechanism of acquired resistance to checkpoint blockade therapy.

Apart from antigen loss, other immunoediting mechanisms such as defects in antigen processing and presentation (58) or in pathways involved in interferon receptor signaling (59) may give rise to acquired resistance to therapy. Gao et al. reported that melanoma patients failed to respond to anti-CTLA-4 therapy due to the loss of IFN-γ signaling caused by genomic defects, such as loss-of-function mutations in JAK1/JAK2 or copy-number alterations in IFN-γ pathway genes (60). In another recent study, pre-treatment and relapse samples from melanoma patients subjected to anti-PD-1 blockade therapy were analyzed to identify resistance-associated mutations. The results showed clonal selection of loss-of-function mutations in JAK1 and JAK2 in two patients, which led to lack of response to interferon gamma, and a truncating mutation in the antigen-presenting protein B2M in another case, resulting in decreased immune cell recognition of tumor cells (61). Moreover, vaccines can increase tumor-infiltrating CD8^+^ T cells that secrete IFNγ, leading to upregulation of the PD1–PDL1 pathway and other inhibitory pathways (62) and creating a negative feedback loop that can suppress tumor immunity.

## Outlook

In the past few years, driven by novel mechanistic insights into cancer immunology and data from clinical trials with checkpoint blockers, tumor neoantigens came into focus in cancer immunology. It became obvious that targeting neoantigens can improve antitumor immunity and minimize off-target toxicities. However, several issues need to be addressed in order to fully harness the power of cancer immunotherapy by targeting neoantigens. First, and most important, considerable research efforts are required to identify the rules that govern the immunogenicity of neoantigens. The majority of the experimentally verified neoantigens that induce antitumor responses are from passenger genes, likely due to the large fraction of passenger mutations (roughly about 90%) compared to driver mutations. Major drawback for developing computational tools for predicting immunogenicity of neoantigens is the dearth of available data. As of today, there are probably few hundred doublets (HLA-neoantigens) and about a dozen triplets (HLA-neoantigens-αβTCR sequences) available for training. Thus, novel medium-to-high-throughput methods are required to generate large enough datasets for data-driven modeling. Second, improved computational methods need to be developed to accurately predict class-II MHC binding neoantigens. Again, major limitation is the limited availability of both positive and negative training data sets. And third, one almost completely unexplored area are neoantigens that are post-translationally modified and the impact of these epitopes on the antitumor immunity. Efforts are underway to tackle these challenges and we will very likely witness in near future exciting developments and discoveries, which will ultimately result in benefit for an individual patient.

## Author Contributions

All authors contributed to the writing of the manuscript.

## Conflict of Interest Statement

The authors declare that the research was conducted in the absence of any commercial or financial relationships that could be construed as a potential conflict of interest. The reviewer MV and handling editor declared their shared affiliation.
